# Front-Line Bevacizumab plus Chemotherapy with or without Maintenance Therapy for Metastatic Breast Cancer: An Observational Study by the Hellenic Oncology Research Group

**DOI:** 10.3390/curroncol29020105

**Published:** 2022-02-17

**Authors:** Stefania Kokkali, Emmanouil Saloustros, Dimitra Stefanou, Paris Makrantonakis, Nikolaos Kentepozidis, Ioannis Boukovinas, Nikolaos Xenidis, Panagiotis Katsaounis, Alexandros Ardavanis, Nikolaos Ziras, Athina Christopoulou, George Rigas, Kostas Kalbakis, Nikolaos Vardakis, Christos Emmanouilides, Ilias Athanasiadis, Athanassios Anagnostopoulos, Dora Hatzidaki, Efthimios Prinarakis, Foteini Simopoulou, Athanasios Kotsakis, Vassilis Georgoulias

**Affiliations:** 1 1st Department of Medical Oncology, Saint Savas Anticancer Hospital, 11522 Athens, Greece; stefaniakokkali8@gmail.com (S.K.); dimitroulastef@hotmail.com (D.S.); ardavanis@yahoo.com (A.A.); 2 Department of Oncology, University Hospital of Larissa, 41221 Larissa, Greece; esaloustros@yahoo.gr; 3 2nd Department of Medical Oncology, Theageneio Anticancer Hospital, 54639 Thessaloniki, Greece; pmakrant@gmail.com; 4 Department of Medical Oncology, 251 Airforce General Hospital, 11525 Athens, Greece; kentenik@hotmail.com; 5 Medical Oncology, Bioclinic Clinic, 54124 Thessaloniki, Greece; ibouk@otenet.gr; 6 Department of Medical Oncology, University General Hospital of Alexandroupolis, 68100 Alexandroupolis, Greece; nxenidis@gmail.com; 7 1st Department of Medical Oncology, Metropolitan General Hospital, 11522 Athens, Greece; pvkatsaounis@gmail.com; 8 2nd Department of Medical Oncology, Metaxas’ Anticancer Hospital, 18537 Piraeus, Greece; zirasngr@otenet.gr; 9 Medical Oncology Unit, Saint Andrew Hospital, 26221 Patras, Greece; athinachristo@hotmail.com; 10 Medical Oncology Unit, General Hospital of Volos, 38222 Volos, Greece; grigas1@yahoo.gr; 11 Department of Medical Oncology, University Hospital of Heraklion, 71500 Crete, Greece; konkalbakis@yahoo.gr (K.K.); nivardak@gmail.com (N.V.); 12 Department of Oncology, Interbalkan Medical Center, 54639 Thessaloniki, Greece; chrem@interbalkan-hosp.gr; 13 2nd Department of Medical Oncology, MITERA Hospital, 11522 Athens, Greece; iliasathanasiadis40@gmail.com; 14 Medical Oncology Unit, E. Dynan Hospital, 11522 Athens, Greece; athanasios.anagnostopoulos@yahoo.gr; 15 Department of Medical Oncology, Hellenic Oncology Research Group, 11471 Athens, Greece; dorachat@med.uoc.gr (D.H.); eprinarakis@horg.gr (E.P.); 16 Department of Radiation Oncology, Iaso Thessaly Hospital, 41005 Larissa, Greece; foteinisim@hotmail.gr

**Keywords:** metastatic breast cancer, bevacizumab, maintenance, real-life data

## Abstract

Front-line bevacizumab (BEV) in combination with taxanes offers benefit in progression-free survival (PFS) in metastatic breast cancer (mBC). The medical records of mBC patients, treated with front-line BEV-based chemotherapy, were retrospectively reviewed in order to generate real life safety and efficacy data. Patients with human epidermal growth factor receptor 2 (HER2)-negative mBC treated with front-line BEV in combination with chemotherapy were eligible. Maintenance therapy with BEV and/or hormonal agents was at the physicians’ discretion. Among the 387 included patients, the most common adverse events were anemia (61.9%, mainly grade 1), grade 3/4 neutropenia (16.5%), grade 1/2 fatigue (22.3%), and grade 1/2 neuropathy (19.6%). Dose reductions were required in 164 cycles (7.1%) and toxicity led to treatment discontinuation in 21 patients (5.4%). The median PFS and the median overall survival (OS) were 13.3 (95% CI: 11.7–14.8) and 32.3 months (95% CI: 27.7–36.9), respectively. Maintenance therapy, with hormonal agents (ET) and/or BEV, was associated with longer OS versus no maintenance therapy (47.2 versus 23.6 months; *p* < 0.001) in patients with hormone receptor (HR)-positive disease and BEV maintenance offered longer OS versus no maintenance in patients with HR-negative disease (52.8 versus 23.3; *p* = 0.023). These real-life data show that front-line BEV-based chemotherapy in HER2-negative mBC patients is an effective treatment with an acceptable toxicity profile. The potential benefit of maintenance treatment, especially ET, is important and warrants further research.

## 1. Introduction

Breast cancer is the most common malignancy and the second leading cause of cancer-related death among women worldwide [1]. Over the past two decades, an improvement in overall survival of breast cancer patients has been noticed. Despite these advances, metastatic breast cancer (mBC) remains a rarely curable disease with a median overall survival (OS) of 24–36 months for the human epidermal growth factor receptor 2 (HER2)-negative subset of patients.

For many years, the medical treatment of HER2-negative mBC was reliant solely on endocrine and multiple cytotoxic agents. Over the past 20 years, treatment has evolved to a more target-directed approach based on the better characterization of the biological underpinnings of breast cancer, and the major pathways involved in tumor progression and metastasis.

Angiogenesis is one of the hallmarks of cancer and vascular endothelial growth factor (VEGF) is the major regulator of angiogenesis in normal and malignant tissues [2,3]. Bevacizumab (BEV) is a humanized monoclonal antibody targeting all VEGF-A isoforms, which prevents the binding of VEGF to its receptors on vascular endothelial cells leading thus, to the inhibition of tumor growth [4]. When combined with a taxane, BEV has shown clinically meaningful activity in first line setting according to phase III studies [5,6,7]. Moreover, additional pre-clinical and clinical data provide evidence of improved efficacy of maintenance or beyond progression anti-angiogenetic therapy in mBC after discontinuation of chemotherapy [8,9].

Real-world data are valuable tools for a more thorough drug benefit–risk ratio evaluation. Safety and efficacy of the drug are studied in a broader population, representing the real clinical setting. Despite the fact that BEV has been tested in several randomized phase III trials, real-life data on mBC are limited. Therefore, we conducted a retrospective observational study in order to evaluate the safety and efficacy of BEV in combination with chemotherapy as first line treatment in HER2-negative mBC patients aiming to fill the gap between clinical trials and daily clinical practice.

## 2. Materials and Methods

### 2.1. Eligibility Criteria

Patients with histologically documented HER2-negative mBC, treated with front-line BEV-containing regimens were enrolled in this observational study. Additional eligibility criteria were: age > 18 years; Eastern Cooperative Oncology Group (ECOG) performance status (PS) of 0–2; adequate hematologic, hepatic and renal function (hemoglobin > 9.5 g/dL; absolute neutrophil count > 1500/μL; platelets > 150,000/μL; total bilirubin < 1.5 times the upper normal limit; and serum creatinine < 2.0 mg/dL) as well as measurable disease according to RECIST criteria 1.1 [10]. Patients were not allowed to have received prior ET or chemotherapy for metastatic disease. Patients with central nervous system (CNS) metastatic disease, who were previously treated with radiotherapy and were neurologically stable, were allowed to participate in the study, whereas patients under therapeutic anticoagulation, regular non-steroidal anti-inflammatory medication, and aspirin (>325 mg/d) were excluded. The institutional review board of each participating institution approved the study protocol, which is registered under the NCT01978977 identifier at the clinicaltrials.gov website [1st Department of Medical Oncology, S. Savas Anticancer Hospital, Athens, 0013-5/4/2011; Department of Oncology, University Hospital of Larissa, Larissa, 431-11/11/2011; 2nd Department of Medical Oncology, Theageneio Anticancer Hospital, Thessaloniki, 8923-8/11/2011; Department of Medical Oncology, 251 Airforce General Hospital, Athens, 9023-11/11/2011; Medical Oncology, ‘Bioclinic’, Thessaloniki, 243-26/10/2011; Department of Medical Oncology, University General Hospital of Alexandroupolis, Alexandroupolis, 10234-14/11/2011; 1st Department of Medical Oncology, Metropolitan General Hospital, Athens, 630-10/11/2011; 2nd Department of Medical Oncology, Metaxas’ Anticancer Hospital, Piraeus, 11980-8/11/2011; Medical Oncology Unit, St. Andrew Hospital, Patras, 4459-24/11/2011; Medical Oncology Unit, General Hospital of Volos, Volos, 183-20/11/2011; Department of Medical Oncology, University Hospital of Heraklion, Crete, 12346-14/2/2011; Department of Oncology, Interbalkan Medical Center, Thessaloniki, 7600-30/11/2011; 2nd Department of Medical Oncology MITERA Hospital, Athens, 987-25/11/2011; Medical Oncology Unit, E. Dynan Hospital, Athens, 2510-25/10/2010; Hellenic Oncology Research Group, Athens; Greece; Responsible Party; Study Sponsor; Department of Radiation Oncology, Iaso Thessaly Hospital, Larissa, Greece, 110-28/12/2010]. The study was conducted in compliance with Good Clinical Practice Declaration of Helsinki and patients gave written informed consent.

### 2.2. Study Treatment and Patients’ Evaluation

The regimens used in the current study consisted of BEV 10 mg/kg every 2 weeks or 15 mg/kg every 3 weeks in combination with either single agent (mainly paclitaxel weekly) or polychemotherapy (also potentially including taxanes) regimens, depending on the physician’s preference. Chemotherapy discontinuation and dose modifications were implemented according to the clinical practice of each center. Maintenance therapy with BEV and/or hormonal agents (endocrine therapy, ET) was optional. If chemotherapy was discontinued due to toxicity, BEV could be continued as maintenance treatment according to physicians’ judgment. Physical examination and blood pressure measurement, toxicity assessment, hematological and biochemical tests were performed before each cycle of treatment according to the clinical practice of each center. Tumor response assessment [mainly by computed tomography (CT) scans] was performed every 3 cycles or earlier if clinically indicated until documentation of disease progression (PD).

### 2.3. Endpoints and Statistical Considerations

Due to the observational nature of the study, no formal hypothesis was applied and no formal sample size calculation was performed. The main objectives of the study were to evaluate the safety (using the NCI Common Terminology Criteria for adverse events, version 4.0) of BEV-based front line chemotherapy in mBC patients using real-life data, as well as to assess the overall response rate (ORR), defined as the proportion of patients with complete (CR) or partial response (PR), the progression-free survival (PFS), defined as the time interval between the date of enrolment, and the date of documented disease progression or death from any cause whichever occurred first and the OS, defined as the time interval between the date of enrolment and the date of death from any cause.

Summary tables (descriptive statistics) and/or frequency tables were provided for all baseline and efficacy variables, as appropriate. Continuous variables were presented with descriptive statistics (n, median, and range). Qualitative factors were compared by Pearson’s chi squared test. Two-sided 95% confidence interval was provided where appropriate. Time-to-endpoint events (PFS, OS) were estimated using the Kaplan–Meier method and the comparisons between several factors were computed with the log-rank test. Median follow-up was calculated using the reverse Kaplan–Meier method. The effect of maintenance treatment on PFS and OS was also examined by Cox’s proportional hazards model and hazards ratios with 95% confidence intervals were provided. All statistical tests were two-sided and *p*-values < 0.05 were considered statistically significant. All clinical data were held centrally (Clinical Trial Office, HORG 11471 Athens, Greece ) and were analyzed using the SPSS statistical software, version 22.0 (SPSS Inc., Chicago, IL, USA).

## 3. Results

### 3.1. Study Population

From 2011 to 2014, 387 patients from 28 centers were included in this analysis. The patients’ median age was 59 years (range: 30–87). The majority of patients had visceral metastases (72.4%), while 41 (10.6%) had only bone disease. Thirteen (3.4%) patients had central nervous system (CNS) metastases, which were previously treated with whole-brain palliative irradiation and were neurologically stable before enrolment. A total of 67.4% of patients had hormone receptor (HR) positive tumors, whereas triple-negative tumors accounted for 25.6% of the cases. A total of 207 patients (53.4%) had received prior adjuvant or neo-adjuvant chemotherapy. Patients’ characteristics are summarized in Table 1.

### 3.2. Treatment Exposure

The vast majority of the patients (93.5%) received BEV in combination with a taxane- based chemotherapy. A total of 2302 treatment cycles (BEV in combination with chemotherapy) were administered with a median of six cycles/patient (range, 1–22). At chemotherapy discontinuation or completion, 306 (79.6%) patients had non-progressive disease [disease control (DC)], 104 (34%) of them received BEV as maintenance therapy, 29 (9.5%) BEV plus ET, and 57 (18.6%) ET alone. Treatment delay was required in 164 cycles (7.1%), due to hematological (27 cycles) and non-hematological (26 cycles) toxicity, or other reasons. Dose reduction was required in 164 cycles (7.1%), mainly due to hematologic toxicity, while treatment was discontinued in 21 patients (5.4%) due to adverse events; BEV discontinuation only was required in 11 patients due to BEV-associated hemorrhage (*n* = 4 patients), pulmonary embolism (*n* = 2 patients), scarring complications including disruption of surgical wound (*n* = 4 patients) and unknown reason (*n* = 1 patient). Among the 321 patients who progressed after front-line treatment, 208 (64.8%) received second-line chemotherapy (187 chemotherapy alone, 21 both second-line chemotherapy and ET), while 34 (10.6%) received only ET systemic treatment.

### 3.3. Safety

The most common adverse events for BEV in combination with chemotherapy were anemia (61.9% in total, mainly grade 1) and neutropenia (grade 3/4 in 16.5% of patients) (Table 2). Fatigue was reported by 23.6% of patients (mainly grade 1/2) and neuropathy by 20.6% (mainly grade 1/2). BEV-attributed side effects were recorded in a small percentage of patients and were grade 1 or 2, including hemorrhage, pulmonary embolism, epistaxis, wound complications (in 2 of them BEV was discontinued due to disruption of a surgical wound), hypertension (4.4% of patients), and proteinuria (2.5% of patients). Bowel perforation occurred in one patient (Table 2). There were four treatment-related deaths, due to pulmonary embolism (*n* = 2 patients), heart attack (*n* = 1 patient), and neutropenic sepsis (*n* = 1 patient).

### 3.4. Efficacy

**Figure 1:**
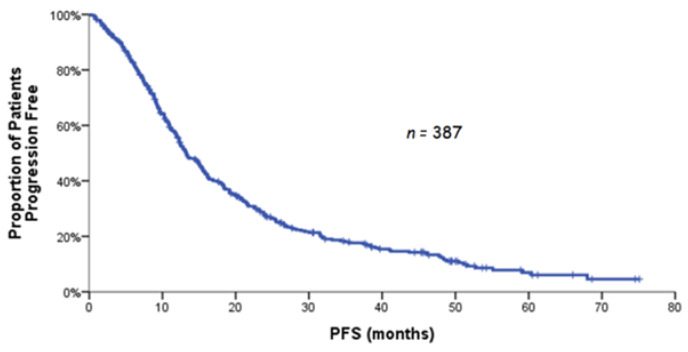
Kaplan–Meier curve for PFS.

**Figure 2:**
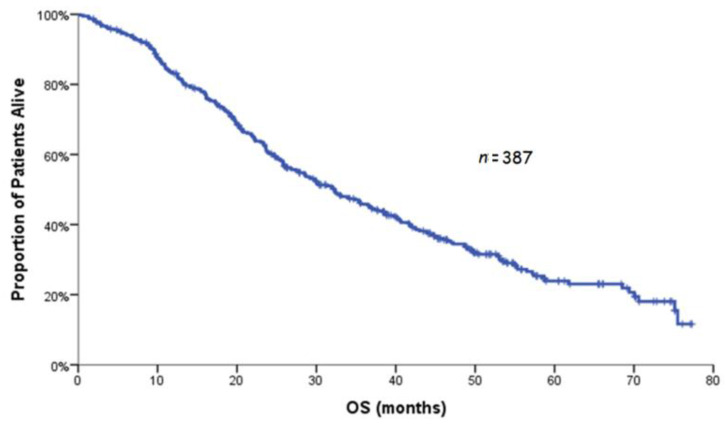
Kaplan–Meier curve for OS.

After a median follow-up of 53.6 months (range: 0.2–77.3), the median PFS was 13.3 months (range: 0.2–75.1; 95% CI: 11.7–14.8), and disease progression at the time of analysis was documented in 321 (82.9%) patients (Figure 1). The median OS was 32.3 months (95% CI: 27.7–36.9; Figure 2). Of 361 evaluable patients, 223 (61.8%) responded to treatment with BEV-based chemotherapy with 2.8% of them achieving CR and 59.0% PR; in addition, 95 patients (26.3%) experienced stable disease (SD) reaching, thus, a clinical benefit rate of 88%. The median duration of response was 12.4 months (range: 0.2–72.6; 95% C.I: 9.6–15.2).

**Figure 3:**
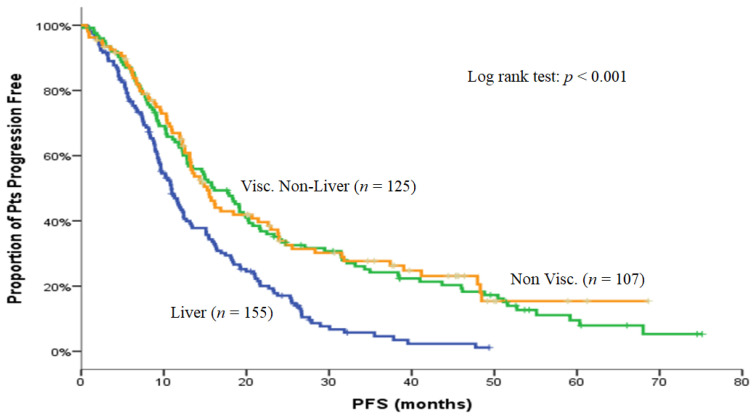

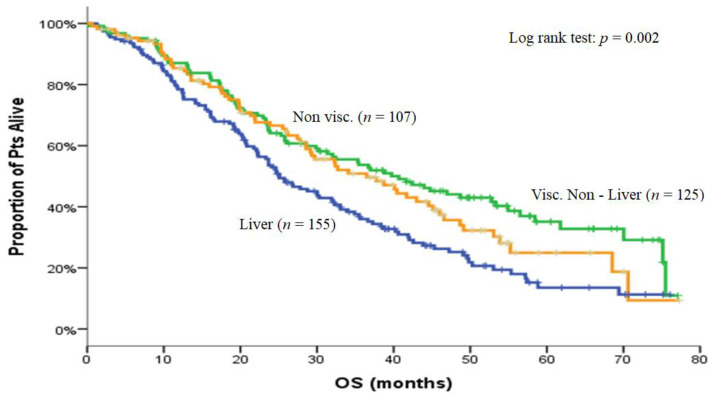
Kaplan–Meier curve for PFS and OS in patients according to visceral-liver, visceral non-liver, and non-visceral metastases.

In order to investigate whether efficacy of BEV was different according to chemotherapy backbone, a planned subpopulation analysis was carried out. There was no difference between taxane-based combination regimens and taxane monotherapy regarding median PFS (13.3 versus 13.2 months; *p* = 0.714), median OS (32.3 versus 35.0 months; *p* = 0.364), and ORR (65.4% versus 59.7%; *p* = 0.289). Moreover, there was no difference in terms of efficacy (ORR, PFS and OS) according to the used taxane (paclitaxel or docetaxel), the age (<70 versus >70 years), or the histologic subtype except for the better ORR in the younger patients (64.6% versus 51.3%; *p* = 0.032). Patients with HR positive disease had a significant higher OS versus those with both HR negative disease [35.5 (95% C.I: 28.9–42.1) versus 25.8 (95% C.I: 20.7–30.9) months, *p* = 0.019)]. A subgroup analysis was also performed according to metastatic site, considering three groups: (i) visceral including liver involvement, (ii) visceral without liver involvement (visceral non-liver), and (iii) non-visceral metastases. Patients with liver metastases experienced the worst median OS [25.1 months (95% C.I: 20.3–29.8) versus 40.7 months (95% C.I: 29.2–52.2) compared with 36.5 months (95% C.I: 26.7–46.2) for patients with visceral non-liver/non-visceral metastases, (*p* = 0.002)]. Similarly, patients with liver metastases had the shortest median PFS [10.9 months (95% C.I: 9.3–12.5) versus 16.1 months (95% C.I: 12.4–19.8) and 15.3 months (95% C.I: 12.8–17.7) in patients with visceral non-liver and non-visceral metastases, respectively, (*p* < 0.001)] (Figure 3).

### 3.5. Maintenance Treatment

**Figure 4:**
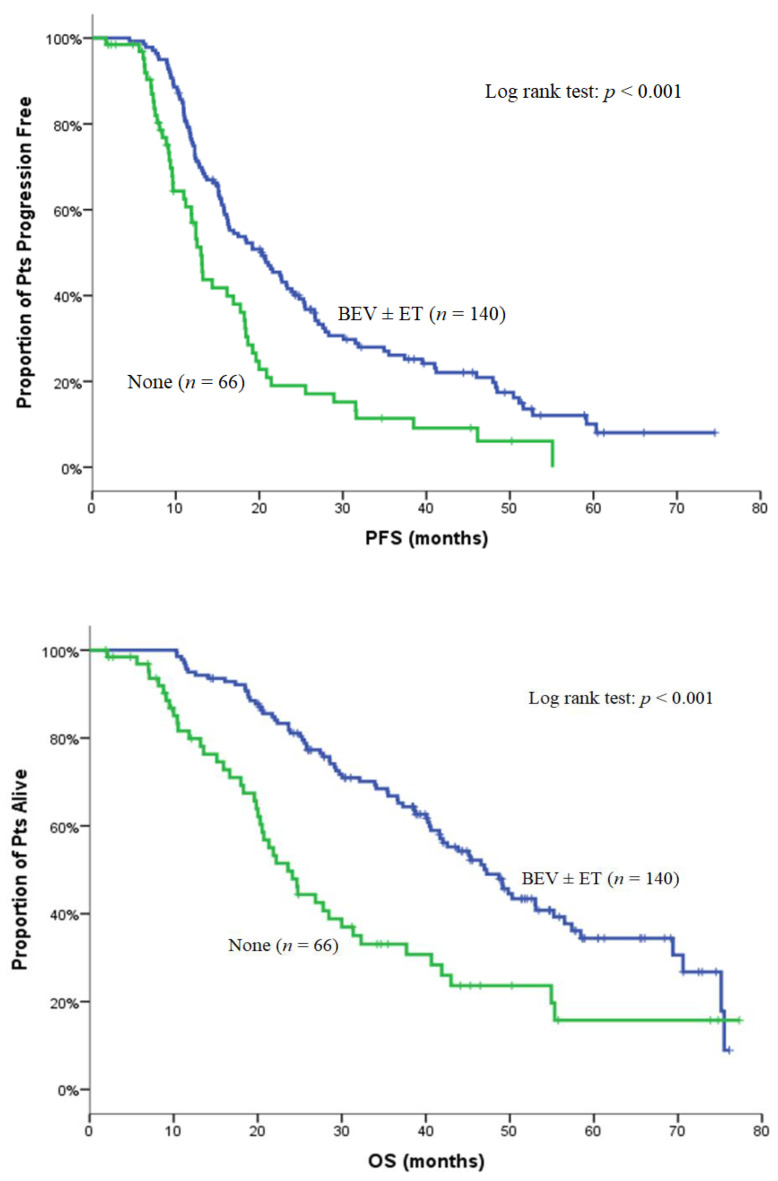
Kaplan–Meier curve for PFS and OS in HR -positive patients without disease progression, according to maintenance treatment (bevacizumab ± endocrine therapy versus no maintenance).

**Figure 5:**
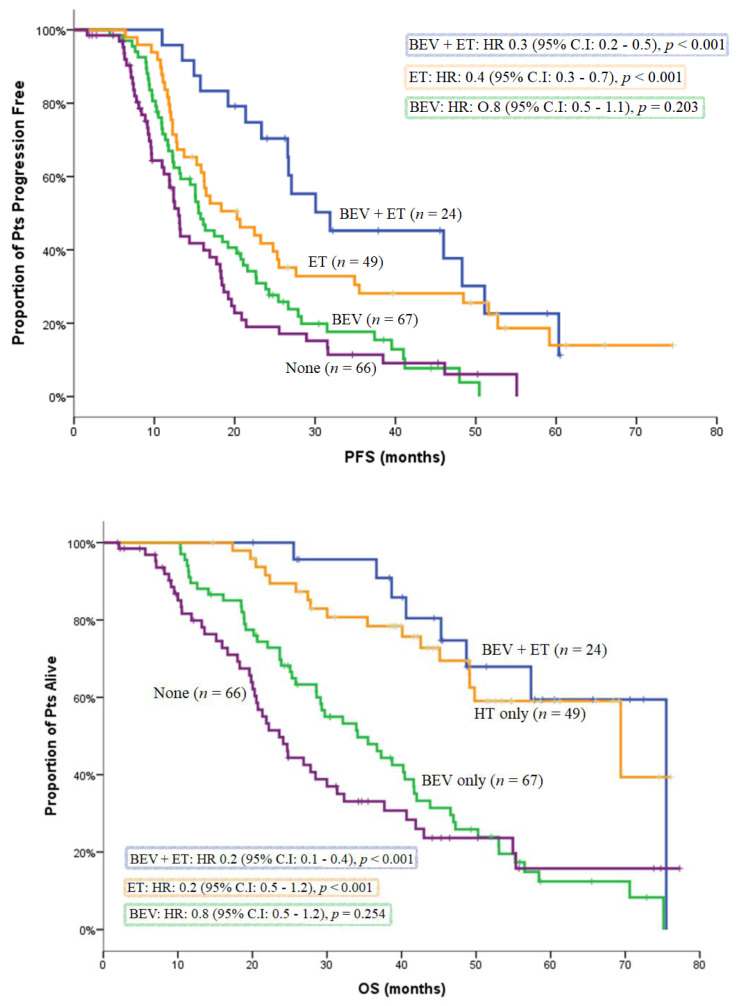
Kaplan–Meier curve for PFS l and OS in HR-positive patients without disease progression, according to maintenance treatment.

Characteristics of non-PD patients who received maintenance treatment with BEV and/or ET are depicted in Table 3. The efficacy of maintenance therapy with either BEV and/or ET was assessed in the subgroup of patients with HR-positive disease without disease progression (*n* = 211). A hundred and forty patients received maintenance treatment with either BEV ± ET or ET alone, while 66 patients did not receive any maintenance therapy. Moreover, five patients from the same non-PD HR-positive group underwent surgery of the primary tumor, or metastasectomy or radiation therapy, and were excluded from maintenance treatment analysis. Clinical outcome was better with maintenance treatment with BEV with or without ET in terms of PFS [20.3 (95% C.I: 15.5–25.1) versus 13.0 (95% C.I: 11.7–14.3) months; *p* < 0.001] and OS [47.2 (95% C.I: 41.0–53.4) versus 23.6 (95% C.I: 19.4–27.8) months; *p* < 0.001] compared to patients who did not receive any maintenance therapy (Table 4 and Figure 4). Moreover, in the same HR-positive group, maintenance treatment with BEV + ET or ET only seems to result in the longest median PFS and median OS. However, this result is not the same for HR-positive patients who received maintenance treatment with BEV only (Table 4, Figure 5). In the HR-negative subgroup, 72 non-progressive patients, 33 patients received BEV as maintenance treatment, and 34 patients received no maintenance therapy, while five patients underwent surgery of the primary tumor or metastasectomy or radiation therapy and were excluded from the maintenance treatment analysis. BEV maintenance was related to a significant improvement of OS [52.8 (95% C.I: 27.0–78.5) versus 23.3 (95% C.I: 17.7–28.8) months; *p* = 0.023), but not of PFS [15.4 (95% C.I: 5.6–25.3) versus 14.6 (95% C.I: 11.8–17.4); *p* = 0.253] (Table 5).

## 4. Discussion

The current study confirmed that patients with HER2-negative mBC treated with chemotherapy combined with BEV in the first-line setting experienced a significant benefit both in terms of PFS and OS. Subgroup analysis showed a more pronounced benefit in patients without visceral metastases, whereas maintenance therapy with BEV in combination with EΤ or EΤ alone was also associated with a better clinical outcome in the HR-positive subgroup. The findings of this study further support the current guidelines for the management of HR-positive mBC, according to which BEV is indicated in combination with taxane or capecitabine as first-line chemotherapy option. In patients with imminent organ failure, the above combination is the optimal first-line therapy, whereas in patients without visceral crisis, cyclin dependent kinase 4 and 6 (CDK4/6) inhibitors combined with ET are the standard-of-care first-line treatment.

Patients treated with BEV-based chemotherapy achieved a similar objective response rate irrespectively of the presence of adverse prognostic features, such as visceral metastases [11,12,13]. However, the presence of liver metastases represents a poor prognostic factor in this setting, as it is associated with worse OS and PFS.

In the current study, longer PFS and OS were reported compared to the majority of the pivotal phase III studies: E2100 (PFS 11.3 months and OS 26.7 months), AVADO (PFS 10 months OS 30.2 months), CALGB (PFS 11 months, OS 27.3 months), and RIBBON1 (PFS 9.2 months, OS not reported) [5,6,7,14,15]. Only IMELDA reported a longer median OS of 39 months in patients receiving BEV in combination to capecitabine [16]. Our results compared favorably to the data from the ESME program, the large-scale French real-life initiative on mBC [17]. In this multi-center study, patients who received paclitaxel in combination with BEV had a significantly higher PFS (HR 0.739; 8.1 months versus 6.4 months) and OS (HR 0.672; median, 27.7 months versus 19.8 months) compared with those who received monotherapy with paclitaxel.

A possible explanation for the survival results in our study may be related to the fact that the number of patients who experienced grade 3–4 toxicity was relatively low mainly due to the weekly administration of paclitaxel as backbone chemotherapy regimen. Therefore, we can expect lower rates of treatment delays or dose modifications compared to previous studies, even if most of these data are not available. In CALGB 40502/NCCTG N063H (Alliance) study, in which patients received weekly paclitaxel at a dose of 90 mg/m^2^, 24% of patients were subjected to dose modifications [14], versus 7.1% in our study. Moreover, the percentage of visceral metastases, which is associated with poor prognosis, was lower in our study compared to other studies (80% in E2100 versus 72.4% in ours) [5,6].

There are also other real-world studies of BEV in combination to chemotherapy in mBC. The multicentric German study AVANTI included >2000 patients who received front-line therapy with BEV in combination to paclitaxel or capecitabine [18]. Median PFS was 12.6 and 10.5 months for the two chemotherapy regimens, respectively, and median OS was 31.4 months. These results are very similar to ours. In addition, in the B-SHARE study, >700 Japanese patients received BEV plus paclitaxel in the first or second line [19]. Median OS was 24.4 months for the patients who received the combination as initial therapy. The presence of visceral metastases was a negative prognostic factor, in accordance with our findings.

The efficacy of BEV in HER2-negative mBC was evaluated in a number of meta-analyses. The combination of BEV plus chemotherapy was found to be superior to chemotherapy alone in terms of ORR and PFS, in a meta-analysis of eight pivotal BEV trials, of which five regarded the first-line setting [20]. Paclitaxel/capecitabine/BEV was reported as the most effective combination in two meta-analyses of BEV in mBC. These analyses included 16 and 20 randomized controlled trials of BEV in different lines of treatment [21,22]. This is in contrast to our analysis, according to which there was no difference in efficacy between taxane monotherapy and taxane-based chemotherapy combinations. Despite the majority of meta-analyses, which indicated a benefit of the addition of BEV, a systematic review of all phase II and III trials of BEV in the first line concluded that the evidence on the use of BEV is not sufficient, based on the lack of significant association between PFS and OS in these trials [23].

Despite the fact that toxicity was acceptable and, as expected from the literature and the safety profile of the used drugs, there were four deaths that have to be considered as treatment related. These deaths underline the need for a close clinical monitoring of patients receiving combination regiments including anti-angiogenic agents. Despite these deaths, there were no other grade 3 and 4 BEV-associated adverse events like hypertension and proteinuria, as already previously reported [12,13]. A large meta-analysis including >6000 patients with mBC treated with BEV identified hypertension, proteinuria, bleeding, cardiac toxicity, and neutropenic fever as BEV-related grade 3–4 adverse events [24]. An interesting observation of our study was that the clinical efficacy of BEV in combination with taxane monotherapy was practically similar, in terms of PFS and OS, with regimens combining taxanes with other cytotoxics and BEV. Taxane partners in combination regimens included liposomal doxorubicin, capecitabine, cyclophosphamide, gemcitabine, or a platinum compound.

The presented data also demonstrate that maintenance treatment with BEV or/and ET may be beneficial for patients. The longest OS was observed in the group of patients that received maintenance treatment combining BEV plus ET with a median PFS of 31.9 months and OS not reached. This observation is in agreement with previous reports [8,25]. Nevertheless, another phase III trial, which evaluated the combination of BEV plus exemestane as maintenance therapy did not reveal any benefit from the combination, but this regimen was compared with the continuation of a regimen including a taxane plus BEV [26]. The combination of BEV with ET has also been studied as first-line therapy and feasibility was demonstrated in a phase II trial of the Sarah Cannon Oncology Research Consortium [27]. The above combination was reported to confer a PFS, but not OS, benefit in the first-line setting, compared to ET monotherapy [28]. Furthermore, age > 65 years, as well as impairment in vision and physical function were revealed as risk factors for toxicity with this combination [29].

In HR-negative patients, maintenance therapy with BEV was associated with a better OS, but not PFS in contrary to what has previously been reported [30]. However, we should mention the relatively small number of patients enrolled in the current study. In contrast, ET as maintenance therapy led to a long both PFS and OS, with a notably high median OS of 69.4 months in HR-positive mBC patients, in accordance with previous findings [30].

Different maintenance strategies have also been reported after induction BEV-based chemotherapy. In the KBCSG-TR1214 trial, the addition of capecitabine to ET maintenance in ER-positive patients led to survival prolongation [31]. Furthermore, in a small phase II trial of triple-negative mBC patients, maintenance therapy with BEV plus erlotinib was administered after nab-paclitaxel plus BEV [32]. A triplet regimen of paclitaxel/capecitabine/BEV, followed by maintenance capecitabine/BEV, was found to be active in triple-negative mBC [33]. All these findings further support the role of maintenance therapy in mBC.

The current report also indicates that strategies extending first line chemotherapy/BEV (>6 cycles versus <6 cycles) were associated with a better clinical outcome in terms of OS, PFS, and RR. Safety and efficacy data were consistent with the findings of previous analyses and meta-analyses [34]. However, there are no data concerning the appropriate duration of the combination of BEV with chemotherapy, as far as the toxicity and the efficacy of the combination is concerned [34]. The Stop & Go study of the Dutch Breast Cancer Research Group (BOOG) also demonstrated that continuous first-line treatment with paclitaxel plus BEV is more efficacious than intermittent treatment, in terms of PFS and OS [35].

Our study has some limitations: (i) patients did not receive a standard chemotherapy regimen, whereas the number of the administered treatment cycles as well as the dose of the drugs were at the discretion of the responsible physician. Therefore, the heterogeneity of the cytotoxic partner and the potential bias may have influenced the results; (ii) it is a single-arm study, without a control arm in order to clearly compare the used treatment with regimens without BEV, and (iii) the number of patients was relatively small in different subgroups.

## 5. Conclusions

In summary, this real-life observational study further confirms previous phase III studies and indicates that the addition of BEV to chemotherapy followed by maintenance BEV is well tolerated and efficacious in patients with HER2-negative mBC. Maintenance treatment with ET and/or BEV resulted in better patients’ outcome. These findings in the real-life setting are complementary to the data from the large randomized phase III trials and reassuring about the use of BEV in association with chemotherapy in the first line setting.

## Figures and Tables

**Table 1:** Demographics and baseline characteristics.

	*N*	%
All patients	387	
Age Median (min–max)	59 (30–87)	
Performance status		
0	311	80.4
1	74	19.1
2	2	0.5
Histology		
Ductal	320	82.7
Lobular	33	8.5
Mixed	6	1.6
Others	28	7.2
Hormone receptors		
At least one positive (ER or PR)	261	67.4
Both (ER and PR) negative	99	25.6
Unknown	27	7.0
Overview to prior treatment		
Prior Surgery	301	77.8
NAC ^1^	13	3.4
Adjuvant chemotherapy	180	46.5
Adjuvant RT ^2^	124	32.0
Palliative RT	16	4.1
Both (adjuvant and palliative RT)	9	2.3
Metastatic sites		
Bone	154	39.8
Liver	155	40.1
Lung + pleura	167	43.1
CNS	13	3.4
Other	200	51.7
Treatment schedules combined with bevacizumab		
Paclitaxel-based	255	65.9
Docetaxel-based	108	27.9
Capecitabine-based	18	4.7
Anthracycline-based	5	1.3
Other	1	0.3

^1^ neoadjuvant chemotherapy, ^2^ radiation therapy.

**Table 2:** Adverse events related to BEV in combination with chemotherapy.

	Gr1		Gr2		Gr3		Gr4	
	*N*	%	*N*	%	*N*	%	*N*	%
Leukopenia	69	17.8	77	19.9	35	9.0	6	1.6
Neutropenia	60	15.5	67	17.3	40	10.3	24	6.2
Anemia	179	46.3	56	14.5	3	0.8	1	0.3
Thrombocytopenia	67	17.3	10	2.6	5	1.3	-	-
Nausea	27	7.0	8	2.1	-	-	-	-
Vomiting	7	1.8	4	1.0	-	-	-	-
Diarrhea	16	4.1	9	2.3	1	0.3	1	0.3
Mucositis	18	4.7	9	2.3	3	0.8	-	-
Constipation	15	3.9	8	2.1	-	-	-	-
Neurotoxicity	38	9.8	38	9.8	4	1.0	-	-
Allergy	14	3.6	3	0.8	-	-	-	-
Edema	11	2.8	5	1.3	-	-	-	-
Skin toxicity	14	3.6	6	1.6	-	-	-	-
Fatigue	56	14.5	30	7.8	5	1.3	-	-
Febrile neutropenia	-	-	-	-	1	0.3	6	1.6
Bleeding (several organs)	67	17.3	6	1.6	-	-	-	-
Bowel perforation	-	-	1	0.3	-	-	-	-
Hypertension	-	-	16	4.1	1	0.3	-	-
Proteinuria	4	1.0	-	-	-	-	-	-
Nail loss	12	3.1	5	1.2			-	-
Hand foot syndrome	4	1.0	1	0.3	-	-	-	-
Conjunctivitis	1	0.3	1	0.3	1	0.3	-	-

Adverse events are graded on a scale from 1 to 4. Gr 1 refers to asymptomatic patients or mild symptoms and Gr 2 to moderate symptoms with minimal, local, or noninvasive intervention indicated. Gr 3 adverse events are severe or medically significant, but not immediately life-threatening and Gr 4 events have life-threatening consequences.

**Table 3:** Characteristics of all patients without disease progression at chemotherapy discontinuation/completion, irrespective of HR status, who received maintenance treatment with BEV and/or ET (*n* = 186).

Characteristic	*N* (%)
Age Median (min–max)	58 (30–82)
Performance status	
0	157 (82.3)
1	29 (15.6)
Histology	
Ductal	153 (82.3)
Lobular	16 (8.6)
Mixed	4 (2.2)
Others	13 (7.0)
Hormone receptors	
At least one positive (ER or PR)	140 (75.3)
Both (ER and PR) negative	33 (17.7)
Unknown	13 (7.0)
Prior treatment	
Prior Surgery	143 (76.9)
NAC ^1^	5 (2.7)
Adjuvant chemotherapy	83 (44.6)
NAC + adjuvant chemotherapy	8 (4.3)
Adjuvant radiation therapy	62 (33.3)
Palliative radiation therapy	6 (3.2)
Both (adjuvant and palliative radiation therapy)	2 (1.1)
Metastatic sites	
Bone	83 (44.6)
Liver	75 (40.3)
Lung + pleura	91 (48.9)
CNS	4 (2.2)
Other	15 (8.1)
Treatment schedules combined with BEV	
Paclitaxel-based	120 (64.5)
Docetaxel-based	57 (30.6)
Anthracycline-based	2 (1.1)
Other	7 (3.8)
Median time of maintenance treatment (min–max) (* Available data for 136 Pts)	7.8 (1.1–70.7)
Maintenance status (*n* = 136)	
Ongoing	11
Toxicity	1
Progressive disease	80
Patients refusal	4
Plateau of stable disease	6
Performance status = 3	1
Complete response	2
Physician’s decision	25
Lost	2
Not available	4

^1^ neoadjuvant chemotherapy.

**Table 4:** Maintenance treatment after completion or discontinuation of bevacizumab-based treatment in HR-positive patients without disease progression. Survival times are measured in months.

	HR-Positive Patients without Disease Progression (*n* = 206)			
Maintenance	*N*	mPFS	HR (95% C.I.)	*p*
No maintenance	66	13.0	reference	
BEV only	67	15.5	0.8 (0.5–1.1)	0.254
ET only	49	20.3	0.4 (0.3–0.7)	<0.001
BEV + ET	24	31.9	0.3 (0.2–0.5)	<0.001
Maintenance	*N*	mOS	HR (95% C.I.)	*p*
No maintenance	66	23.6	reference	
BEV only	67	34.1	0.8 (0.5–1.2)	0.203
ET only	49	69.4	0.2 (0.1–0.4)	<0.001
BEV + ET	24	NE	0.2 (0.1–0.4)	<0.001

HR: hormone receptor, mPFS: median progression-free survival, HR: hazard ratio, mOS: median overall survival, 95% C.I: 95% Confident Interval, BEV: bevacizumab, ET: endocrine therapy, NE: not reached.

**Table 5:** Maintenance treatment after completion or discontinuation of bevacizumab-based treatment in HR-negative patients without disease progression. Survival times are measured in months.

HR-Negative Patients without Disease Progression (*n* = 67)							
PFS				OS			
	BEV Only	None	*p*		BEV Only	None	*p*
*N*	33	34		*N*	33	34	
Median PFS Min–Max 95% CI	15.4 5.1–75.1 5.6–25.3	14.6 1.0–43.7 11.8–17.4	0.253	Median OS Min–Max 95% CI	52.8 5.4–77.1 27.0–78.5	23.3 3.9–68.5 17.7–28.8	0.023

HR: hormone receptor, N: number, PFS: progression-free survival, OS: overall survival, Min–Max: minimum-maximum, 95% C.I: 95% Confident Interval, BEV: bevacizumab.

## Data Availability

The data presented in this study are available on request from the corresponding author.
